# Role of indocyanine green to look for vascularity of the pancreatic stump during Whipple’s procedure and its clinical implications in terms of post-pancreatectomy acute pancreatitis and postoperative pancreatic fistula

**DOI:** 10.1136/bmjsit-2024-000318

**Published:** 2025-06-12

**Authors:** Dhiresh Kumar Maharjan, Prabir Maharjan, Yugal Limbu, Roshan Ghimire, Prabin Bikram Thapa

**Affiliations:** 1 GI and General Surgery, Kathmandu Medical College and Teaching Hospital, Kathmandu, Bagmati, Nepal; 2 Department of GI and General Surgery, Kathmandu Medical College and Teaching Hospital, Kathmandu, Central Development Region, Nepal; 3 Department of GI and General Surgery, Kathmandu Medical College and Teaching Hospital, Kathmandu, Nepal

**Keywords:** Health Technology, Laparoscopy

## Abstract

**Objectives:**

Post-pancreatectomy acute pancreatitis (PPAP) has been a well-defined entity by the International Study Group of Pancreatic Surgery. Underlying cause may be hypoperfusion at remnant stump of pancreas, which has been linked with additional post-pancreatectomy complications like postoperative pancreatic fistula. The primary goal was to assess the vascularity of remnant pancreas utilizing indocyanine green with near-infrared fluorescence. Indocyanine Green could aid in objectively mitigating hypoperfusion status of the pancreatic stump.

**Design:**

Hospital-based descriptive study conducted as per the revised Strengthening the Reporting of Observational Studies in Epidemiology guidelines between 1 August 2022 and 2 August 2023.

**Setting:**

This study was conducted in tertiary care centers of Kathmandu.

**Participants:**

All 43 participants who underwent pancreaticoduodenectomies were included who completed the study.

**Intervention:**

Blood supply to the remnant of the pancreas during pancreaticoduodenectomy was assessed utilizing indocyanine green, capturing distinct arterial, venous, and port venous phases, which were analyzed after 10 to 15 s of administration.

**Main outcome measures:**

In three instances, indocyanine green dye revealed unequal vascular supply at the pancreatic remnant, requiring adjustments to the margins before completing the anastomosis of the remnant pancreas and the jejunum.

**Results:**

PPAP was noticed in eight patients (18.6%), among which five patients (11.6 %) had postoperative hyperamylasemia, and three had grade B PPAP. The outcomes revealed that in the 40 patients with adequate perfusion, PPAP occurred in seven patients(16.3%), and grade B clinically relevant postoperative pancreatic fistula occurred in one patient. In contrast, among the three patients with inadequate perfusion, after revision of the pancreatic margin, PPAP was observed in one patient, and none of them had clinically relevant post-operative pancreatic fistula.

**Conclusion:**

Postoperative acute pancreatitis, ultimately exhibiting the possibility of postoperative pancreatic fistula, must be monitored with vigilance. While several elements contribute to fistula formation, ensuring sufficient vascular supply at the pancreatic remnant using indocyanine green may alleviate presumed PPAP and associated complications. The dye could aid in enhancing surgical outcomes following pancreaticoduodenectomy.

**WHAT IS ALREADY KNOWN ON THIS TOPIC**

- It is a known entity that the hypovascular pancreatic margin is a risk factor for post-pancreatectomy acute pancreatitis and postoperative pancreatic fistula.

**WHAT THIS STUDY ADDS**

- Indocyanine green fluorescence is an innovative tool for identifying pancreatic margins with adequate vascularity.

**HOW THIS STUDY MIGHT AFFECT RESEARCH, PRACTICE OR POLICY**

- The study acknowledges using indocyanine green (ICG) as an adjunct to the objective assessment of the pancreatic margin. Similar studies and applications of ICG can pertain to assessing margins of upper gastrointestinal, hepatobiliary, and colorectal anastomosis.

## Introduction

 Pancreatoduodenectomy (PD) is among the most complex surgical interventions in hepato-pancreatico-biliary surgery, primarily indicated for malignant lesions of the pancreatic head, ampulla, or duodenum. Despite advances in surgical techniques and perioperative care, the procedure still carries a notable morbidity rate as high as 60% and a mortality rate of about 2% to 10%.^1^

Acute pancreatitis of the remnant pancreas has gained significant attention among surgeons globally. Elevated serum and drain amylase levels following pancreatectomy have been observed, leading to the development of the concepts of postoperative hyperamylasemia (POH) and post-pancreatectomy acute pancreatitis (PPAP). This framework emphasizes the association between POH, PPAP, and postoperative pancreatic fistula (POPF) with PD-related complications, highlighting PPAP as an emerging clinical entity that can significantly contribute to prolonged hospital stays, escalating healthcare costs, and adversely affecting patient outcomes. Clinically relevant postoperative pancreatic fistula (CR-POPF) varies within the range from 6% to 14%.^2^,^4^

The incidence of PPAP has been proportioned directly to CR-POPF, which has been attributed in terms of multiple determinants, featuring the patient’s overall condition, the texture of the pancreatic parenchyma, and the size of the main pancreatic duct (MPD), including the surgeon’s expertise.^35^,^7^

Moreover, complex microvascular perfusion of pancreatic parenchyma around the neck region by the superior, inferior right branch, and left branches of the dorsal pancreatic artery, after transection during PD, might bring about hypoperfused watershed area of the pancreatic stump. This may be the reason for triggering inflammation of pancreatic parenchyma, which is further aggravated by hypoperfusion of the remnant pancreas, leading to pathological shunts and hyperperfusion injury, leading to pancreatic necrosis and POPF.^8 9^ Hence, with the assistance of indocyanine green (ICG) imaging, macrovascular blood supply during the arterial phase and microvascular perfusion of parenchymal remnant can be objectively visualized.

Building on these observations, our objective was to assess the effectiveness of ICG fluorescence in detecting sufficient perfusion at the pancreatic remnant in patients following PD and its clinical implications regarding PPAP or POPF.

## Methods

This study included all patients who underwent PD for 1 year between 1 August 2022 and 2 August 2023, operated by the same team of surgeons in two hospitals: Kathmandu Medical College Teaching Hospital and Nepal Cancer Hospital.

All the participating patients provided their written consent for the study according to the Declaration of Helsinki. Ethical clearance was procured from the Institutional Review Board of Kathmandu Medical College Teaching Hospital (Ref:02022025/01)

### Study design and endpoints

This hospital-based descriptive study was conducted per the revised Strengthening the Reporting of Observational Studies in Epidemiology guidelines.^10^ The primary goal was to assess the vascularity of the remnant pancreas utilizing ICG with near-infrared (NIR) fluorescence.

Our secondary endpoints were to examine how these findings correlated with the incidence of PPAP with the incidence of CR-POPF. POH, PPAP, and CR-POPF were defined and classified based on the International Study Group of Pancreatic Surgery (ISGPS) guidelines.^11^

The surgical team comprised a professor and an associate professor, each with substantial experience in pancreatic procedures, performing approximately 50 such surgeries per year. This level of expertise ensured consistency in surgical technique and minimized operator-dependent variability. By integrating real-time ICG fluorescence assessment, the study sought to illuminate potential perfusion deficits at the pancreatic remnant, which may influence the development of PPAP and eventually result in CR-POPF.

### Surgical technique

All patients underwent staging laparoscopy followed by either laparoscopic resection, converted to open, or straight open PD. Those with borderline and locally advanced malignancy received neoadjuvant chemotherapy, and only this subset of patients underwent the triangle operation. A standard PD was performed invariably, in which a radical tumor resection was performed with complete en bloc removal of the intervening connective tissue. This technique was designed to minimize residual disease and optimize oncological outcomes. The transection of the pancreas was identified along the axis of the superior mesenteric and the main portal vein.

Reconstruction of the pancreatic remnant predominantly involved a pancreatico-jejunostomy (PJ) anastomosis using Blumgart’s technique. Postoperatively, the incidence of PPAP and CR-POPF rate was documented as per the ISGPS guidelines.^11^

### Indocyanine green (ICG) dosing

**Figure 1:**
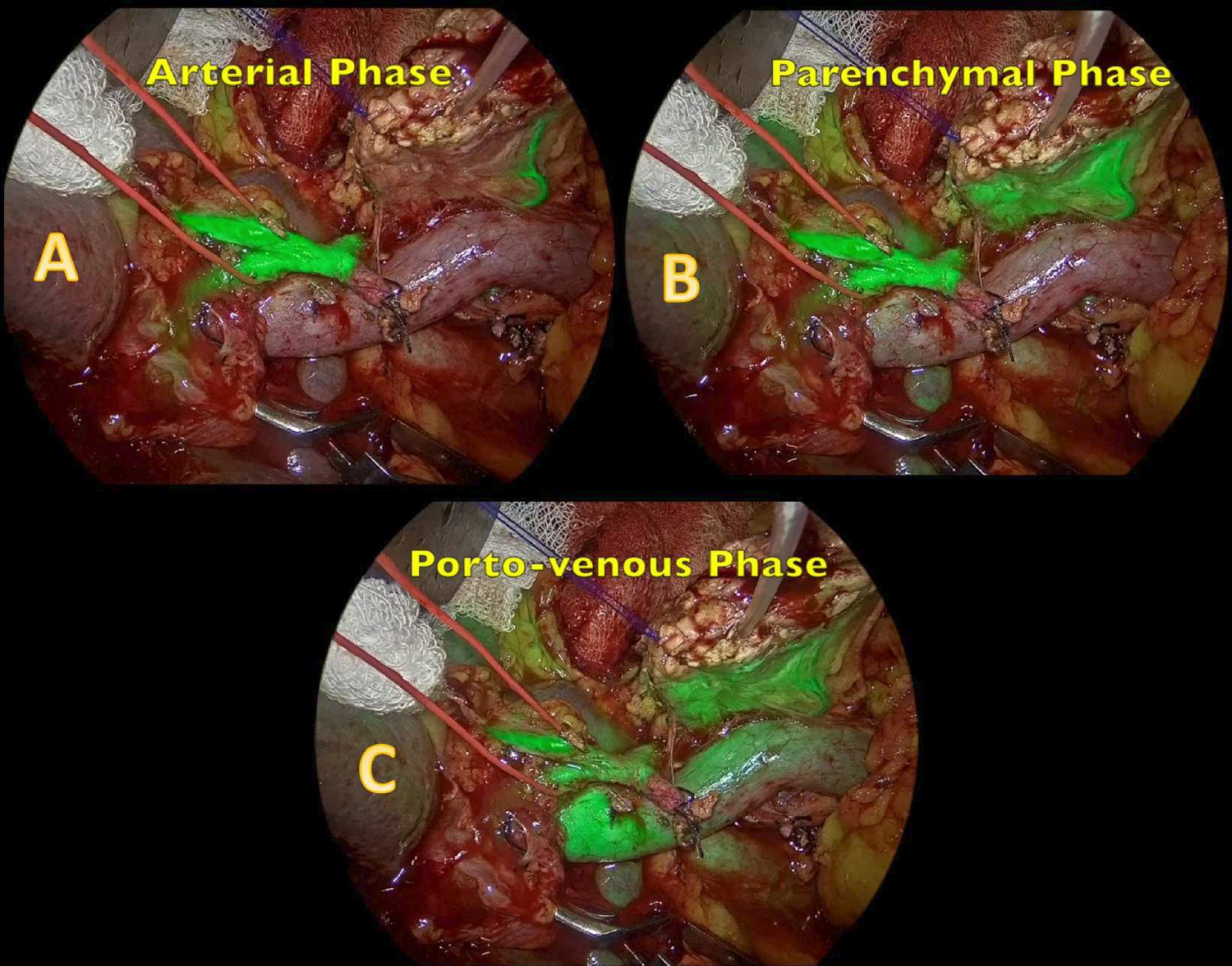
ICG NIR imaging phases: A, arterial phase showing the hepatic artery; B, pancreatic phase showing the pancreas with heterogeneous uptake of the resected margin; and C, porto-venous phase. ICG, indocyanine green; NIR, near-infrared.

The powder of ICG (25 mg) was dissolved with 10 mL of normal saline, resulting in a 2.5 mg/mL concentrated mixture. A 3 mL dose of this solution (7.5 mg of ICG) was delivered intravenously, with a simultaneous 10 mL saline flush. NIR imaging was employed to visualize ICG fluorescence.^12^ Imaging phases were analyzed in real time after seconds of administration as follows:

- Arterial phase (common hepatic artery, celiac artery, and superior mesenteric artery): The first signs of fluorescence were observed for approximately 1–2 s, reflecting the arterial supply through major abdominal arteries (figure 1A).
- Pancreatic arterial phase (pancreatic stump): Fluorescence in the pancreatic arterial system, specifically at the pancreatic stump, was observed for approximately 2 s thereafter (figure 1B).
- Venous phase (inferior vena cava): Venous uptake of ICG in the inferior vena cava was observed for another 2 s, coinciding with systemic venous return.
- Porto-venous phase (portal vein, splenic vein, and pancreatic stump): Enhanced fluorescence was noted in the portal vein, splenic vein, and pancreatic stump for 5 s, representing the venous drainage phase through the hepatopancreatic region (figure 1C) (online supplemental video).

Perfusion was considered adequate if the pancreatic stump exhibited homogeneous fluorescence under NIR imaging. Heterogeneous or absent fluorescence indicated hypoperfusion. In cases of hypoperfusion, the pancreatic transection line was revised based on the observed ICG fluorescence to enhance perfusion. After revision, the adequacy of vascular supply at the new margin was re-evaluated using ICG fluorescence before proceeding with the anastomosis.

### Data analysis and statistical analysis

All collected data were entered into a secure electronic database. Key outcomes measured included the frequency of stump margin revisions guided by ICG fluorescence and the incidence of PPAP and CR-POPF. Appropriate statistical methods were employed to evaluate these outcomes and determine their significance. The study population included all the cases who underwent PD. Data were collected with the help of a proforma and entered in SPSS V.21. Categorical variables were presented as absolute or relative frequencies with percentages, while continuous variables were demonstrated as mean±SD

## Results

During this period of 1 year, 43 patients underwent PD. The study showed 60.5% of the total participants were female, with a median age group of 68±4 years. The age of patients was divided into two categories, with 30 patients (69.8%) being younger than 70 years and 13 patients (30.2%) being 70 years and above. The majority of the patients (31, 72.1%) had a resectable pathology and did not receive any neoadjuvant therapy. There was one patient with chronic pancreatitis with a dominant head mass among this group of patients, while eight patients (18.6%) had a borderline resectable pathology, and the remaining four patients (9.3%) had a locally advanced pathology, who underwent neoadjuvant chemotherapy.

The indications for the surgery varied, as pancreatic head carcinoma accounted for 17 patients (39.5%), followed by ampullary carcinoma in 12 patients (27.9%). Distal cholangiocarcinoma followed the previous two indications, with 10 patients (23.2%). This distribution highlighted the predominance of pancreatic head carcinoma among the surgical cases.

The average duration of surgery was 5.2±1.1 hours, and the average loss of blood was 270±150 mL. The size of the MPD was below 3 mm in 18 patients (41.9%) and more than 3 mm in 25 patients (58.1%). Pancreatic texture was assessed, with 15 patients (34.9%) having a soft pancreas, while 28 patients (65.1%) had a non-soft pancreatic consistency. (table 1)

**Table 1:** The demographics and intraoperative findings of the patients

Variables	Subcategory	Number of patients	Percentage
Gender	Male	17	39.5%
	Female	26	60.5%
Age	<70 years	30	69.8%
	>70 years	13	30.2%
Stage of the disease	Resectable	31	72.1%
	Borderline resectable	8	18.6%
	Locally advanced	4	9.3%
Neoadjuvant	Yes	12	27.9%
Chemotherapy	No	31	72.1%
Indications of surgery	Pancreatic head carcinoma	17	39.5%
	Ampullary carcinoma distal	12	27.9%
	Cholangiocarcinoma duodenal	10	23.2%
	Adenocarcinoma pancreatic	2	4.6%
	Neuroendocrine tumor	1	2.3%
	Chronic pancreatitis (dominant head mass)	1	2.3%
Duration of surgery	Mean±SD (hours)	5.2±1.1	
Intraoperative blood loss	Mean±SD (mL)	270±150	
Pancreas duct size	Less than 3 mm	18	41.9%
	More than 3 mm	25	58.1%
Pancreas consistency	Soft	15	34.9%
	Not soft	28	65.1%
ICG use	Homogenous perfusion	40	93.1%
	Heterogenous perfusion	3	6.9%

ICG, indocyanine green.

### Postoperative outcomes

The incidence of PPAP was noted in eight patients (18.6%). PPAP was further subclassified into POH in five patients (11.6%) and grade B PPAP in three patients (6.9%). Among them, a biochemical leak was observed in three patients, whereas grade B POPF was observed in one patient. None of them had grade C POPF.

The association between perfusion adequacy and POPF-related outcomes revealed that in the 40 patients with adequate perfusion, PPAP developed in seven patients (16.3%), and CR-POPF Grade B occurred in one patient. In contrast, among the three patients with inadequate perfusion, after revision of the pancreatic margin, Grade B PPAP was observed in one patient, and none of them had CR-POPF.

Delayed gastric emptying (DGE) was identified in five patients (11.6%). When subclassified by severity, Grade A DGE occurred in three patients (6.9%), Grade B DGE was noted in two patients (4.7%), and no cases of Grade C DGE were reported. Postoperative complications, graded according to the Clavien-Dindo classification, were distributed, and Grade 1 complications were seen in 15 patients (34.9%), Grade 2 complications came about in 10 patients (23.3%), Grade 3a complications were observed in two patients (4.6%), and Grade 4a complications were recorded in one patient (2.3%) (table 2).

**Table 2:** Postoperative results of the patients who underwent PD

Variables	Subcategory	Number of patients	Percentage
Clavien-Dindo Classification	Grade 1	30	69.70%
	Grade 2	10	23.30%
	Grade 3a	2	4.60%
	Grade 4a	1	2.30%
PPAP	POH	5	11.60%
	Grade B	3	6.90%
	Grade C	none	
CR-POPF	Biochemical leak	3	6.90%
	Grade B (with Grade B PPAP)	1	2.30%
	Grade C	None	0.00%
DGE	Grade A	3	6.90%
	Grade B	2	4.60%
	Grade C	0	0.00%

CR-POPF, clinically relevant postoperative pancreatic fistula; DGE, delayed gastric emptying; PD, pancreatoduodenectomy ; POH, postoperative hyperamylasemia; PPAF, post-pancreatectomy acute pancreatitis.

## Discussion

**Figure 2:**
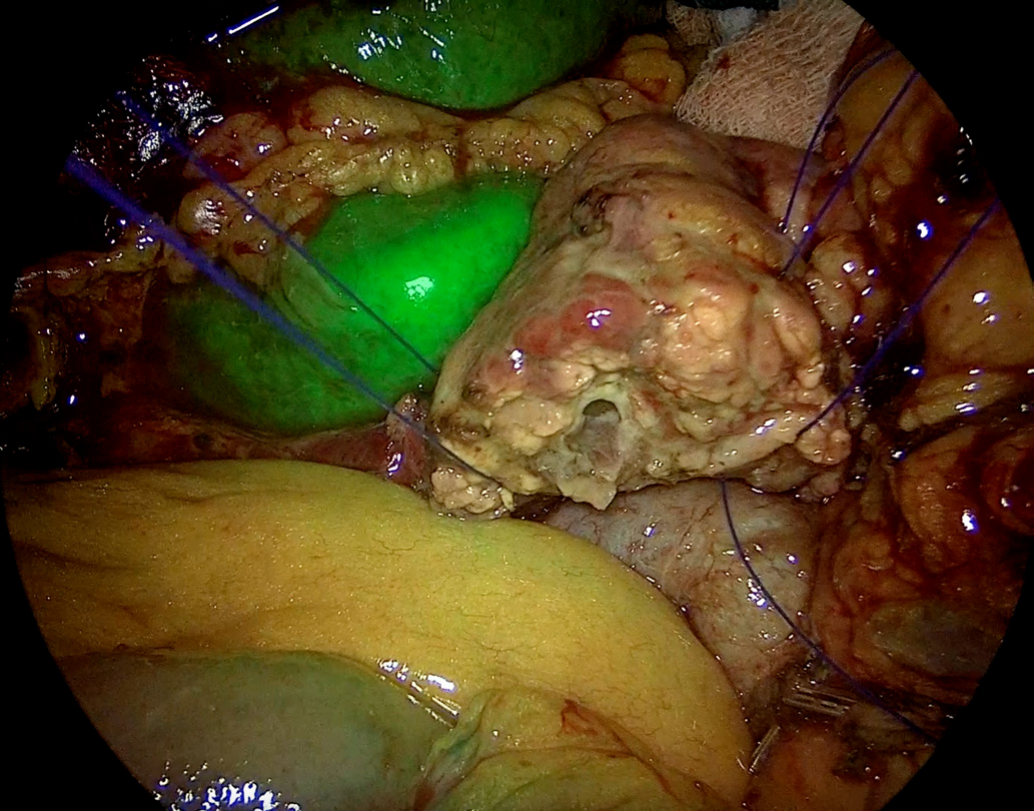
Pancreatic stump margin showing absent ICG uptake. ICG, indocyanine green.

Fluorescence imaging with ICG has become a key tool in visceral surgery in recent periods. ICG is considered a potential intraoperative tool for the assessment of the anastomotic stumps before and after the reconstruction. ICG is gaining popularity due to its easy availability, short half-life, and immediate intraoperative response. ICG can be utilized to evaluate both arterial and venous perfusion and provide an instant breakdown of the functionality of the microvascular anastomoses when used with an integrated operating microscope system.^13 14^ Even though visual assessment of vascularity is common, ICG imaging allows for more precise identification and documentation of microvascular stability.^12^ In our study, in three cases, though the naked eye appeared normal, heterogeneous perfusion appeared on ICG imaging. Hence, the remnant pancreas was refashioned towards the distal end before the reconstruction (figures2^4^). The potential to detect microvascular perfusion confirms good vascularity intraoperatively at the pancreatic remnant, mitigating the incidence of PPAP and CR-POPF. Moreover, CT perfusion data, including arterial flow and mean transit time, has shown a correlation with the occurrence of PPAP and POPF in PD.^15^,^18^

**Figure 3:**
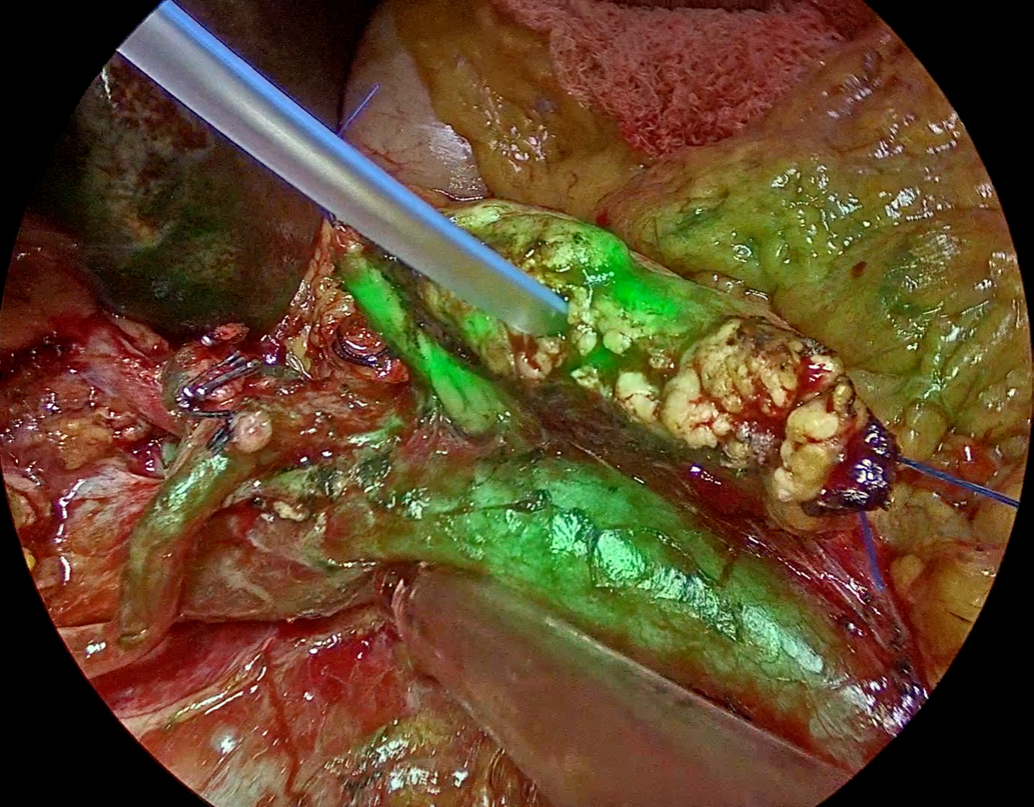
Heterogeneous ICG uptake of pancreatic stump. ICG, indocyanine green.

**Figure 4:**
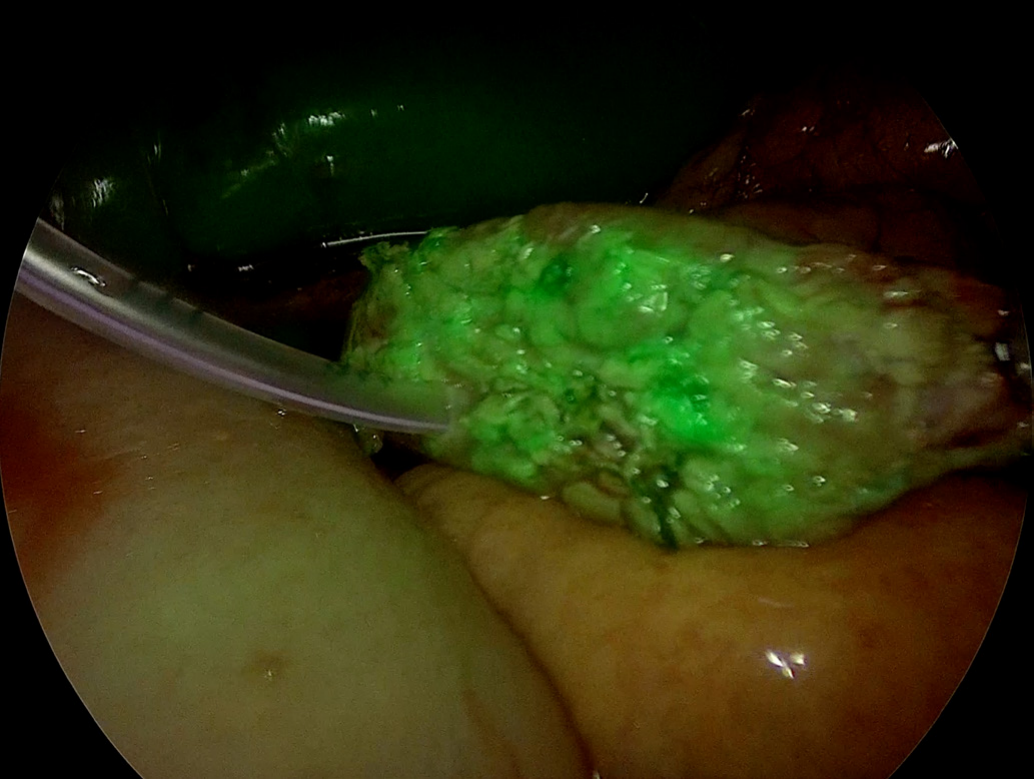
Pancreatic stump margin showing adequate perfusion.

A systematic review by Robertson *et al* included five studies encompassing over 156 patients with a comprehensive overview discussing diverse perfusion assessment methods, including ICG. The review stated a higher overall incidence of hypoperfusion (35%) and subsequent management strategies, like stump margin revision. The authors also stated a 12% incidence of POPF with 0.6% mortality and, similarly, 1% of PPH. However, the review included a lot of variability considering definitions, methodologies, and heterogeneity across studies.^14^ In our study, 40 out of 43 (93.02%) were found to have sufficient perfusion, and the remaining three (6.98%) necessitated amendment of the remnant pancreas (figures1^4^). All pancreatico-enteric anastomoses relied on ICG fluorescence.

Similarly, a cohort conducted by Chen *et al* included 27 out of 37 patients who underwent robotic PD and were assessed for pancreatic stump hypoperfusion. The authors stated a high correlation between hypoperfusion of the pancreatic stump and CR-POPF, 67% in the hypoperfusion group, and 17% were in the non-hypoperfused group (p=0.026).^15^ In our study, among three patients who underwent refashioning of the pancreatic stump, only one patient had PPAP, while none of them developed Grade B or C POPF.

However, observations made by Chen *et al* focused on the impact of suture-induced hypoperfusion as one of the reasons impacting the incidence of CR-POPF. Our study acknowledges the need for an objective assessment of the microcirculation of the remnant pancreas, for which ICG shows its potential.

The Strasberg study has highlighted the significance of sufficient vascularity in the pancreas border during PD.^9 13 16^ According to Strasberg *et al*, 62% of the patients in this study exhibited adequate vascular supply at the pancreatic remnant on visual assessment. The transection of the pancreatic neck was along the superior mesenteric and portal vein axis. The occurrence of CR-POPF declined in patients when the pancreatic margin was reformed to the distal end to obtain satisfactory fluorescence before the reconstruction. Busnardo *et al* demonstrated a hypovascular region situated between the isthmus and the body of the pancreas, along these two sections. The significance of the watershed area was highlighted in this study.^17^

Even though the literature supports the utilization of ICG as an adjunct, some studies differ as well. In 2018, a study done by Rho *et al* studied the utility of ICG in achieving complete resection in pancreatic head adenocarcinoma and demonstrated better clearance of the retroperitoneal resection margins with no considerable differences in mean operative duration, loss of blood, and POPF-associated complications. Although the pancreatic texture and the size of the MPD are largely unchangeable, ischemia at the resection margin can be prevented, which aids in reducing the chance of CR-POPF. The pancreatic neck is considered a watershed area, with vascular supply originating from both the head and body, resulting in a region of relative ischemia that might result in anastomotic failure.^19 20^

The Visualizing Ischemia in the Pancreatic Remnant) is an ongoing phase II clinical trial that focuses on demonstrating the efficiency of intraoperative ICG of the resected pancreas to correlate with postoperative fistulas. The findings of this trial will contribute remarks on the benefit of ICG measurements in projecting CR-POPF.^21^

An interesting correlation between CR-POPF and PPAP has been an area of discussion. Imaging, a cornerstone for diagnosing non-surgical acute pancreatitis, proves less effective in the postoperative context. CT, while a valuable tool, often fails to capture subtle edematous changes associated with mild PPAP. This diagnostic gap risks underestimating clinically significant PPAP, delaying timely intervention. These limitations have catalyzed a shift toward a more nuanced diagnostic approach, prioritizing biochemical, clinical markers, and intraoperative adjuncts over radiological reliance.^22^,^24^ Holmberg *et al* conducted a retrospective observational analysis of over 1000 patients, where they tackled the challenge in diagnosing and managing PPAP. The author focused on serum amylase and CRP as tools to predict PPAP and CR-POPF. Our study emphasizes on the advantage of the ICG as a predictor for inadequate perfusion of the pancreatic stump, which might lead to PPAP and anastomotic failures, eventually leading to POPF.^22^ Doussot *et al* uniquely explored PPAP, identifying a correlation between hypoperfusion and local pancreatic inflammation. The study demonstrated the utility of ICG in identifying hypoperfused pancreatic stump areas and correlating to PPAP and, eventually, CR-POPF risk. Higher serum amylase and lipase levels and radiological confirmation of PPAP offer potential early markers for complications, adding another dimension to ICG’s role in mitigating risks. Recently, the clinical validation of ISGPS for the definition of PPAP has enlisted the criteria of POH or hyperlipasemia on the first and second postoperative days with radiological alterations and clinically relevant deterioration, which has directly influenced the outcomes of CR-POPF. Thus, ICG as adjuncts to prevent such complications seems eminent.^24^,^28^

There were some limitations to the current study, which included data from a single surgical team from two centers. To overcome these limitations, further multicenter, prospective, and randomized controlled trials should be performed.

## Conclusion

The relationship addressed between inadequate perfusion, PPAP, and subsequent CR-POPF underscores the importance of integrating ICG as a valuable resource in assessing and documenting the adequacy of the pancreatic stump vascular supply during PD, which can help minimize the morbidity of the patient. Incorporating these approaches could enhance early detection and intervention, ultimately improving patient outcomes after surgery.

## Supplementary material

10.1136/bmjsit-2024-000318 online supplemental file 1

## Data Availability

Data are available upon reasonable request.
